# Clinical and regulatory perspectives on biosimilar therapies and intended copies of biologics in rheumatology

**DOI:** 10.1007/s00296-016-3444-0

**Published:** 2016-02-27

**Authors:** Eduardo Mysler, Carlos Pineda, Takahiko Horiuchi, Ena Singh, Ehab Mahgoub, Javier Coindreau, Ira Jacobs

**Affiliations:** Reumatólogo en Organización Médica de Investigación, Buenos Aires, Argentina; Instituto Nacional de Rehabilitacion, Mexico City, Mexico; Kyushu University Beppu Hospital, Beppu, Japan; Pfizer Inc., Collegeville, PA USA; Pfizer Inc., 235 East 42nd Street, New York, NY 10017-5755 USA

**Keywords:** Biosimilar, Intended copy, Biologic, Pharmacovigilance, Regulatory

## Abstract

Biologics are vital to the management of patients with rheumatic and musculoskeletal diseases such as rheumatoid arthritis and other inflammatory and autoimmune conditions. Nevertheless, access to these highly effective treatments remains an unmet medical need for many people around the world. As patents expire for existing licensed biologic (originator) products, biosimilar products can be approved by regulatory authorities and enter clinical use. Biosimilars are highly similar copies of originator biologics approved through defined and stringent regulatory processes after having undergone rigorous analytical, non-clinical, and clinical evaluations. The introduction of high-quality, safe, and effective biosimilars has the potential to expand access to these important medicines. Biosimilars are proven to be similar to the originator biologic in terms of safety and efficacy and to have no clinically meaningful differences. In contrast, “intended copies” are copies of originator biologics that have not undergone rigorous comparative evaluations according to the World Health Organization recommendations, but are being commercialized in some countries. There is a lack of information about the efficacy and safety of intended copies compared with the originator. Furthermore, they may have clinically significant differences in formulation, dosages, efficacy, or safety. In this review, we explore the differences between biosimilars and intended copies and describe key concepts related to biosimilars. Familiarity with these topics may facilitate decision making about the appropriate use of biosimilars for patients with rheumatic and musculoskeletal diseases.

## Introduction

A biologic medicine is a large molecule derived from living cells and typically produced by recombinant DNA, hybridoma, or other technologies [1]. Biologics are used in the treatment, diagnosis, or prevention of several non-communicable and some communicable diseases and conditions and include hormones, small proteins, vaccines, monoclonal antibodies, and fusion proteins [2]. The introduction of biologics (e.g., etanercept, adalimumab, infliximab, rituximab, abatacept, and tocilizumab) revolutionized treatment algorithms in patients with rheumatic and musculoskeletal diseases (RMDs), including chronic autoimmune inflammatory diseases, such as rheumatoid arthritis, psoriatic arthritis, ankylosing spondylitis, and juvenile idiopathic arthritis [3–7]. These conditions incur high individual, societal, and health care system costs [5] and have a significant effect on patient health-related quality of life and risk of comorbidities [8]. Achieving remission in the clinical and functional domains of rheumatic diseases has become a feasible target with biologic therapies [4], and treatment should be focused on achieving this target or, at a minimum, low disease activity in every patient [5]. However, the escalating burden of chronic diseases, including RMDs, and worldwide shortage of rheumatologists amid cost constraints and national health care system heterogeneity constitute barriers that have resulted in limited access to biologics. Today, access to these highly effective treatments for rheumatic diseases remains an unmet medical need for many people around the world [9].

As patents expire for existing licensed (referred to as reference or originator) biologics, biosimilar products can be approved by regulatory authorities, and thus enter clinical use. Biopharmaceutical manufacturers around the world are developing versions of the existing licensed biologics; however, not all of these versions have evidence to demonstrate comparable physiochemical and functional properties, efficacy, and safety profiles as defined by regulatory authorities and recommended by the World Health Organization (WHO) [10]. This is of particular interest to rheumatologists as many biopharmaceutical manufacturers worldwide are developing follow-on biologics of products used in the treatment for rheumatologic conditions, including adalimumab, etanercept, infliximab, and rituximab.

Biosimilars are highly similar copies of originator biologics approved through defined and stringent regulatory processes after having undergone rigorous analytical, immunogenicity, non-clinical, and clinical comparative evaluations. Non-comparable biotherapeutic products (also known as “intended copies” or “biomimics”) are copies of originator biologics that have not undergone such evaluations and have not met strict regulatory requirements such as those of the WHO, European Medicines Agency (EMA) [11], or US Food and Drug Administration (FDA) [12]. In this review, we explore the differences between biosimilars and intended copies and discuss the unique developmental processes used for biologics, including biosimilars. We also summarize the regulatory standards for the development and approval of biosimilars in various regions of the world and examine current differences between regions. Further, we review several key areas of interest for biosimilars, including naming, pharmacovigilance, and risk management.

## Biosimilars

The term “biosimilar” is a regulatory definition that refers to a biologic product that is developed to be highly similar to, and treat the same conditions as, an existing licensed or approved biologic product. A biosimilar, as defined by the WHO, is a “biotherapeutic product which is similar in terms of quality, safety, and efficacy to an already licensed reference biotherapeutic product,” in which similarity is defined as the “absence of a relevant difference in the parameter of interest” [10]. Biosimilars are highly similar versions of marketed biologic medicines and are supported by appropriate analytical and immunogenicity testing and non-clinical and clinical trials to demonstrate that they are sufficiently “similar” in quality, efficacy, and safety to their reference (originator) biologics. Unlike conventional medicines, also called “small molecules,” biologic drugs are large, structurally complex molecules. Because they are biologics, biosimilars should not be viewed as generic medicines, and unlike small-molecule generics, they cannot be manufactured to be identical to the originator biologic. Manufacturing of biosimilars is generally more complex than manufacturing generics. In addition, the regulatory process for biosimilar approval is very different from the approval process for small-molecule generic medicines, and it is precisely the regulatory process that defines these categories. Although both biosimilars and generics require pharmacokinetic bioequivalence studies, biosimilars must demonstrate high similarity to the originator product [10–12], whereas small-molecule generics must show proof of quality (i.e., identical chemical structure).

## Non-comparable biotherapeutic products (intended copies)

Prior to the implementation of science-based regulatory pathways for the approval of biosimilars, copies of originator biologic products were introduced in some countries. The basis for approval of these copies has not been clear as they lack comparative studies to an appropriate reference product. Most, if not all of these products, can be considered as non-comparable biotherapeutic products, or intended copies (sometimes referred to as biomimics [13, 14]). Intended copies of biologics can be defined as copies of already licensed biologic products that have not met the requirements of the WHO, EMA, or FDA to establish biosimilarity [10–12]. In other words, intended copies are products for which the manufacturer intended to make a copy but did not follow a comparative development pathway with the reference medicine. Their similarity exercises may be incomplete, analytical evidence may be insufficient, or they may either lack clinical trials or were studied only in limited or methodologically inadequate clinical trials [15, 16]. Often, intended copy products are developed independently and are not directly compared against a licensed biologic product, and they may or may not be compared clinically. Thus, the data available to assess intended copies do not provide adequate comparable efficacy and safety to the licensed product. These products may have clinically significant differences in formulation, dosages, efficacy, or safety from what is required for a biosimilar [17]. There is no clear evidence that intended copies have efficacy and safety similar to the originator biologic or a biosimilar owing to the absence of rigorous clinical testing.

It is important that rheumatologists distinguish between intended copies and biosimilars. In some countries without biosimilar regulations or with non-stringent regulatory environments, intended copies are being approved as generic drugs, which allows pharmaceutical manufacturers to make and sell copies of the reference drug without establishing proper biosimilarity between these products and the reference products [18, 19]. Furthermore, in some countries with less stringent regulation, copies of biologics have been marketed without clinical trials [20] or based on studies that were limited in scope, size, or scientific rigor [17]. For example, intended copies of etanercept are marketed in several countries, including China, India, Colombia, and Mexico. Additionally, an intended copy of rituximab is manufactured in India and marketed in India and several Latin American countries [19, 21] despite the apparent lack of a comparative clinical trial with the originator biologic in patients with RMDs. Because data are lacking to establish that these products are highly similar to the originator biologic, they cannot be considered to be biosimilars [17]. Compounding this problem is the challenge of finding detailed study methods and results for these copies in the public domain (e.g., clinical trial registries, published congress abstracts, and indexed publications) that would permit independent evaluation of products. This is in sharp contrast to the monoclonal antibody CT-P13 (Remsima™/Inflectra™), approved by EMA and under review by the FDA as a biosimilar to reference infliximab (Remicade^®^). In analytical studies, CT-P13 demonstrated an identical amino acid sequence as the originator infliximab, production on the same type of cell line, comparable pharmacodynamics, including binding activity to human tumor necrosis factor alpha (TNFα), and cytotoxic activities against a cell line expressing transmembrane human TNFα [22, 23]. This detailed in vitro characterization was followed by clinical evaluations that demonstrated comparable efficacy, safety, and immunogenicity to originator, infliximab [22, 24]. Although in Europe these findings were not part of the product label, the study methods and results were made available through European public assessment reports (EPAR) and peer-reviewed publications [22–24].

## Manufacturing biologics

In order to appreciate the need for science-based regulatory pathways for approval of biosimilars and the differences between biosimilars and intended copies, it is important to understand the molecular complexity of biologics and how they are manufactured. Important differences exist in the molecular complexity and manufacturing processes between small-molecule drugs and biologics. As their name implies, small-molecule drugs are small in size and have less complex chemical structures. The chemical structure of a generic small-molecule drug can be characterized using current technology to guarantee that it is identical to the active drug in the reference product [25]. In contrast, biologics are proteins typically developed using living systems, such as bacteria, yeast, or mammalian cells, and are much larger and more complex than small-molecule drugs [26]. Protein structure and manufacturing processes to produce them have been discussed extensively in the literature [27–32] and will not be provided in detail in this manuscript. The pharmacologic action of monoclonal antibodies used to treat RMDs will depend not only on the amino acid sequence (primary structure) [25, 33], but also on secondary, tertiary, and, in some cases, quaternary structures, which are referred to as higher-order structures [34, 35]. Because of the relationships between structure and function, clinically meaningful differences in safety, purity, and/or potency may result from modifications of primary or higher-order structure [2]. Some biologics are glycosylated. Changes in the pattern of carbohydrates attached to the amino acid chain (i.e., glycosylation pattern), which can result from production in living cells, may occur during manufacture based on the type of cells used to produce the biologic and the multistep manufacturing process. Because the glycosylation pattern may also contribute to the clinical profile of the biologic, changes in the pattern may also alter clinical outcomes [25, 36].

The manufacturing process for biologics is substantially more specialized than that for small-molecule drugs. The creation of a cell line used in the manufacturing of biologics involves numerous intricate processes, including isolation of a targeted gene sequence, cloning of that gene sequence, and the use of a DNA vector to transfer the targeted DNA into an expression system [33]. During production, the cell line goes through a series of fermentation processes. The protein of interest is then harvested, purified, formulated, and packaged. There is a strong relationship between the manufacturing processes and the characteristics of the final product, which are very sensitive to production conditions [37]. Changes or differences in manufacturing processes may have a significant impact on the quality, purity, biologic characteristics, and clinical activity of the final product [26, 33, 38].

Manufacturers of biologics should have sophisticated, state-of-the-art manufacturing technology and quality management systems that meet all necessary regulatory requirements [39]. The manufacturing process for the originator biologic product is proprietary. Therefore, the biosimilar developer must analyze the reference product extensively and reverse engineer to produce a biologic agent that is highly similar to the reference product, which requires substantial knowledge, experience, and expertise regarding the development and manufacture of biologics.

### Comparability of biologic proteins

The inherent complexity of biologics and the intricacies of the manufacturing process make the final product sensitive to changes in production conditions. Subtle changes in, or differences between, manufacturing processes may result in significant differences that can impact quality, purity, efficacy, safety, and immunogenicity [26, 38, 40].

Over time, manufacturers of originator biologics may implement manufacturing process changes for their molecules for reasons such as improving product stability, increasing the scale of production, or complying with changes in regulatory requirements [25, 41]. Manufacturers of originator products must evaluate their products pre- and post-manufacturing change to ensure there are no differences in efficacy or safety. In 1996, the FDA established the concept of “comparability” in the published guidance document, Demonstration of Comparability of Human Biological Products, including Therapeutic Biotechnology-derived Products [41]. Subsequently, this concept was included in the International Conference on Harmonisation (ICH) guidance Q5E, Comparability of Biotechnology/Biological Products Subject to Changes in Their Manufacturing Process [42]. Comparability can be based on analytical testing (e.g., monomer/aggregate levels, charge heterogeneity), in vitro biologic assays (e.g., cell-based bioassays), and/or non-clinical and clinical data [42, 43]. The scientific principles described in ICH Q5E also apply to the development of biosimilars; however, manufacturers of biosimilars use different cell lines, raw materials, equipment, processes, and process controls than the originator. Therefore, in general, the amount of data needed to establish similarity will be greater than what is required to determine comparability following an originator’s manufacturing change [12].

Nevertheless, it is important to recognize that highly sophisticated analytical technologies are the basis for establishing both similarity and comparability. Biologics have become increasingly well characterized, and biosimilars are specifically engineered and designed to closely resemble the originator molecule to the best extent possible using current technologies. Sensitive state-of-the-art orthogonal analytical methods are typically able to assess similarity with high confidence [44]. Early in product development, small differences (e.g., in epitope or binding characteristics of a biosimilar monoclonal antibody) would be identified from the extensive physicochemical and functional characterization conducted for biosimilars.

## Regulatory pathways for biosimilars

**Fig. 1:**
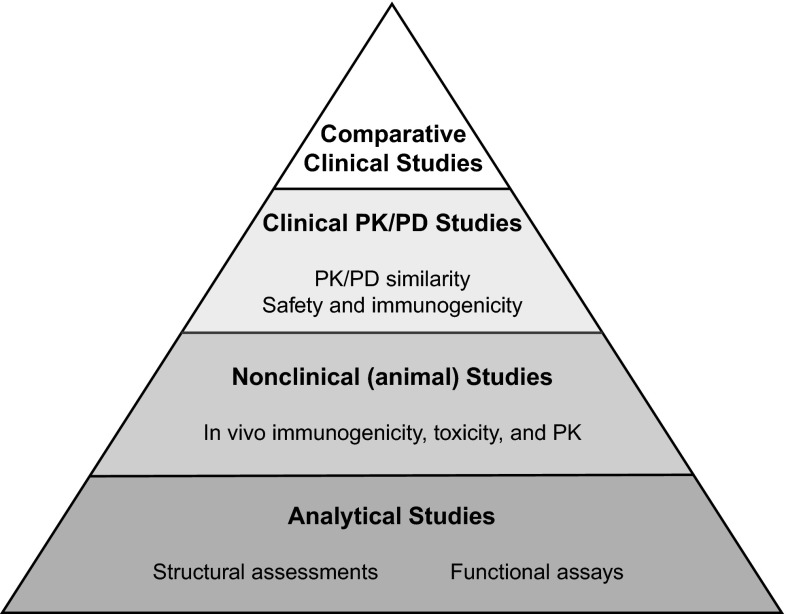
Biosimilar development process. Strong evidence of biosimilarity during analytical and non-clinical studies is essential. The objective of a biosimilar program was to establish biosimilarity, and the clinical program is focused and tailored toward this objective. Adapted from McCamish and Woolett [41], https://creativecommons.org/licenses/by-nc/3.0/us/legalcode

Regulatory requirements for approval of biosimilars are generally consistent across the EMA, Health Canada, and WHO, and the guidelines issued by the FDA [10–12, 45]. Although minor differences exist among these agency guidelines, with some slight differences in terminology, all require a stepwise approach to establish biosimilarity (Table 1). Biosimilars are comparable to authorized biologics with demonstrated similarity to the reference product in terms of structure, function, and biologic activity. These established regulatory pathways include comparative assessments involving analytical, non-clinical, and clinical studies. Regulations require head-to-head comparative studies for structural characterization, functional in vitro assays, pharmacokinetic and pharmacodynamic evaluations, and safety, efficacy, and immunogenicity assessments [10–12]. Biosimilarity is considered demonstrated based on the totality of the evidence from all evaluations, with each step supported by the preceding one (Fig. 1).

**Table 1:** Overview of biosimilar regulatory guidelines

	EMA [11]	WHO [10]	Canada [45]	USA [12, 76]	Japan [60]	Australia [90]
Definition	A biologic medicinal product similar to another biologic medicine that has already been authorized for use	A biotherapeutic product that is similar in terms of quality, safety, and efficacy to an already licensed reference biotherapeutic product	A biologic drug that enters the market subsequent to a version previously authorized in Canada, with demonstrated similarity to a reference product	A biologic product highly similar to the reference product notwithstanding minor differences in clinically inactive components	A biotechnological drug product developed by a different company, which is comparable with an approved biotechnology-derived product	A version of an already registered biologic medicine that has a demonstrable similarity in physicochemical, biologic, and immunological characteristics, efficacy, and safety, based on comprehensive comparability studies
Preclinical data	Target binding; signal transduction, functional activity/viability of cells of relevance. If in vitro comparability is satisfactory, animal studies may not be required	Receptor-binding or cell-based assays; relevant biologic/PD activity, toxicity	Receptor-binding or cell-based assays; Animal PD and repeat-dose toxicity studies, and other relevant safety observations	Structural analyses, functional assays; animal toxicity assessments, PK/PD, immunogenicity (unless determined not necessary by FDA)	Toxicity and pharmacologic assessments, PK, and local tolerance	Similar to EMA
Clinical trials	Comparability demonstrated in stepwise process using PK, PD (if feasible), followed by clinical efficacy and safety trials	PK, PD, (confirmatory PK/PD), efficacy, and safety	PK, PD, clinical efficacy, and safety, including immunogenicity	Study or studies including assessments of immunogenicity and PK or PD	PK, PD (with appropriate PD marker) consider safety studies (immunogenicity)	Similar to EMA
Naming	Commercial name, appearance, and packaging should differ; INN should be the same for related biosimilars	Changes are being considered to the current policy of using INN	Not specified	Draft guidance proposes that all biologics be given a four-letter suffix to the INN	Non-proprietary name of the reference product followed by “BS” and an abbreviation to reference the manufacturer	Australian biologic name without a specific biosimilar identifier suffix (policy is under review)
Pharmacovigilance	Risk management pharmacovigilance plan must be submitted; clinical safety monitored closely after marketing authorization	Pharmacovigilance plan submitted with marketing authorization application; describe planned post-marketing activities	Risk management plan submitted prior to marketing authorization; periodic safety update reports. Serious adverse drug reactions reported within 15 days	Any risk evaluation and mitigation strategy for the reference product applies. Post-marketing studies or additional clinical trials could be mandated	Post-authorization safety studies monitored on a continuous basis	Risk management plan outlining pharmacovigilance procedures to be implemented submitted with biosimilar application

*EMA* European Medicines Agency, *WHO* World Health Organization, *FDA* Food and Drug Administration, *TGA* Therapeutic Goods Agency, *PD* pharmacodynamics, *PK* pharmacokinetic, *INN* International Non-proprietary Name

As a first step, analytical studies are conducted to confirm that the biosimilar has a foundation of quality based on structural and functional similarity to the reference product. Non-clinical studies then demonstrate that the biosimilar agent acts on the same target or physiologic process and has similar toxicity as the reference product. A crucial element in the evaluation of a biosimilar product is a tailored clinical trial program that compares the pharmacokinetics, clinical efficacy and safety, and immunogenicity of the biosimilar with that of the reference product.

### Considerations for comparative clinical trials for biosimilars

Clinical development of a biosimilar necessitates a rigorous head-to-head comparison with the reference product. The goal is to demonstrate that any difference in efficacy or safety between the biosimilar and reference products is less than a pre-specified margin of clinical equivalence [46]. Comparative clinical (phase III) trial designs for biosimilars are similar to those for any biologic with respect to most-sensitive patient population, sample size, end points, and study duration (Table 2) [47]. The trials should be randomized, double-blinded, and adequately powered [48].

**Table 2:** Key considerations in evaluating comparative clinical (phase III) studies of biosimilars [47]

Comparability	An equivalence design at the 90 or 95 % confidence interval is used (generally preferred to a non-inferiority design) An equivalence design establishes that the biosimilar is neither superior nor inferior to the reference product
Patient population	Should be clinically relevant Does the study use the most sensitive patient population, that is, the population in which clinically meaningful differences in safety and effectiveness between the biosimilar and reference product are most likely to be detected?
Power/sample size	Study is sufficiently powered to detect potential differences between biosimilar and reference product
Dose	The dose and route are consistent with the reference product
End points	End points are relevant to the disease state and sensitive enough to detect clinically relevant differences in efficacy and safety, if any, between the biosimilar and reference product
Study duration	The duration of the study was appropriate to detect clinical effects
Statistical analysis	A per-protocol analysis includes only patients who followed the protocol, whereas an intention-to-treat analysis includes all randomized patients If the study used an equivalence design, a per-protocol analysis was used
Efficacy	Are efficacy measures within the pre-specified acceptable margin of equivalence?
Safety	Are the incidence and types of adverse events comparable between biosimilar and reference product?

Because the goal of a comparative clinical trial is to demonstrate that the proposed biosimilar is equivalent to the reference product, superiority trials are not appropriate. Instead, non-superiority trials, including equivalence and non-inferiority designs, are most suitable [12]. Although non-inferiority trials can be used, an equivalence study design is preferred to demonstrate that the biosimilar is equivalent to the reference product. The goal in an equivalence trial is to reject the null hypothesis of non-equivalence and accept the alternative that the biosimilar and reference products are equivalent. In other words, the trial should determine whether the biosimilar is no worse than and also not better than the reference product. This is accomplished using a two-sided test (requiring superior and inferior margin limits) based on a pre-specified equivalence margin, which is selected to detect clinically meaningful differences in effectiveness between the biosimilar and reference product at the 95 % confidence interval [12, 49]. In some situations, a one-sided non-inferiority design may be appropriate if justified (e.g., if the original drug has a wide safety margin) [12, 48]. However, a one-sided test does not demonstrate equivalence, but instead demonstrates that one product is not inferior to another. This leaves open the possibility of a more potent drug, which could produce a higher number of adverse events; however, these studies often do not carry the statistical power to demonstrate this.

Other important considerations in the design of a comparative clinical study between a biosimilar and reference product are sample size, study duration, and end points. Sample size is a key determining factor of the power of a study and may be affected by the treatment effects and the equivalence margins. For example, as the equivalence margins narrow, the minimum sample size increases [12]. The disease or condition for which the biosimilar is being studied will influence the duration of the study. When evaluating a biosimilar for RMDs, many of which are chronic, the comparative clinical trial should be of sufficient duration so that both beneficial clinical effects and potential adverse effects may be observed. Generally, end points are selected based on the end points that were used in the clinical trials of the reference product [12]. Furthermore, for studies in RMDs, end points are most often consistent with the Outcome Measures in Rheumatology (www.omeract.org).

## Evolving regulatory landscape

Regulation of biosimilar products varies among the many countries of Latin America. Although some countries in Latin America have yet to introduce guidance for biosimilars, most are moving toward establishing standards of regulation for these products [50]. In recent years, ANMAT (Administración Nacional de Medicamentos, Alimentos y Tecnología Médica) in Argentina, ANVISA (Agência Nacional de Vigilância Sanitária) in Brazil, and COFEPRIS (Comisión Federal para la Protección contra Riesgos Sanitarios) in Mexico have developed their own regulatory pathways for biosimilar products by combining and customizing WHO and EMA guidelines for biosimilars to address their regional needs [51]. In Brazil, there is a traditional regulatory pathway for new biologic products, which is based on a full dossier presentation. For follow-on biologics, there are two pathways: a comparative pathway, which closely follows the WHO guideline, and an individual pathway, which allows a reduced dossier presentation. Only products licensed by the comparative pathway are considered biosimilars [52]. The Colombian Ministry of Health and Social Protection (Ministerio de Salud y Protección Social de Colombia) [53] also released new draft guidelines for biologics, which includes biosimilars. The proposed draft guidelines outline three routes for registration of biologic products: a complete route, a comparability route, and an abbreviated route, which aims to facilitate the registration of biosimilar products in Colombia through an abbreviated pathway. In addition, in Chile, Costa Rica, Guatemala, Panama, and Peru, recent guidelines have been developed based on or taking into consideration internationally accepted standards for evaluation of biosimilars [54].

Intended copies of biologics have been used in Latin American countries for many years prior to the establishment of regulatory guidelines for assessing similarity between these products and their reference products. Intended copies of several biologics have been approved without quality clinical trials or adequate evaluation and remain on the market in some countries in Latin America (Table 3). For example, in Mexico, until recently, the approval of an intended copy of a biologic drug followed the same criteria as that of a generic small-molecule drug and preclinical and clinical data were not required. By 2011, this policy had resulted in 23 intended copies of biologics registered in Mexico as generics [18]. The Mexican College of Rheumatology in 2012 published a position paper regarding the necessary scientific evidence required to evaluate the efficacy and safety of biosimilar drugs before and after their arrival to the Mexican market [55]. A warning to health professionals was issued by the Mexican Federal Commission for Protection Against Health Risks concerning anaphylactic reactions associated with rituximab when the originator product rituximab and the intended copy Kikuzubam^®^ were interchanged [56]. In March 2014, a health notice was issued requiring the manufacturer recall all batches of Kikuzubam due to safety concerns and, subsequently, the product was withdrawn from the market [57]. How to re-evaluate products that were previously approved but do not fit the country’s current regulatory criteria for a biosimilar is a significant global issue, especially in Latin America, as the WHO recent draft guidelines reflect [18, 58, 59]. It is anticipated that the complete assessment process for these drugs may be required to be completed within 2 years from the effective date of new regulation, with existing dossiers and comparative analytical characterization data being reviewed in the first 8 months. Strict adherence to the new regulation should be mandatory for these drugs including clinical studies.

**Table 3:** Countries in which intended copies of listed biologics for rheumatic conditions are approved and/or marketed without biosimilar regulations [14, 17, 91, 92]

Year of market introduction	Rituximab	Etanercept
2007	India (Reditux™)	–
2008	Peru (Reditux™)	Colombia (Etanar™)
2010	Chile, Bolivia (Reditux™) Mexico (Kikuzubam^®^)^a^	–
2011	Jamaica, Ecuador (Reditux™)	China (Yisaipu)
2012	Paraguay (Reditux™)	Mexico (Etart™; Infitam™)
2013	–	India (Etacept™)

^a^Withdrawn in 2014

A growing number of countries in Asia have established or are establishing regulatory pathways for evaluation and approval of biosimilars. Japan and South Korea released guidelines in 2009 [60, 61], and Singapore and Malaysia have generally followed EMA guidelines [62, 63]. India released official biosimilar guidelines in 2012 [64, 65]; however, these guidelines are viewed by some as less strict than those from the WHO, EMA, and FDA because the potential exists for reduced non-clinical and clinical testing programs if there is proof of strong quality comparability and manufacturing process consistency [19]. It should be noted that before the biosimilars regulatory pathway was issued in India in 2012, more than 25 products designated as “similar biologics” had already been approved [66]. Late in 2014, China’s Center for Drug Evaluation (CDE) published draft guidance for approval of biosimilars. The new draft guidance outlines the principles of comparability and a stepwise approach to testing [67]. Previously, domestic “copy biologic medicines” were produced in China and were approved through their traditional new drug approval process, without comparison to the reference product.

## Biosimilar naming

“Biosimilar” is a regulatory term and is distinct from naming and the WHO International Non-proprietary Name (INN) assignment process for biologics. Although the INN system, first adopted more than 50 years ago, defines global standards for the nomenclature of pharmaceuticals, naming of biosimilars is under discussion in many regions [68–70]. No international harmonization on biosimilar naming exists [71], and this creates challenges because the naming of biosimilars has consequences for pharmacovigilance (the detection, assessment, and prevention of adverse effects after a product is launched onto the market) [26]. Each biosimilar product should be easily identified and distinguished from the reference product and from other biosimilars to ensure traceability and accurate reporting of adverse drug reactions [72]. Having an INN qualifier unique to the specific biosimilar manufacturer would be prudent from a traceability, pharmacovigilance, and safety perspective [69]. Because intended copies often have the same INNs as the reference products, traceability may be problematic or impossible.

The WHO guidelines for naming of biosimilars have advised that non-glycosylated biosimilars share the INN of the reference product, whereas glycosylated biosimilars should have a Greek letter suffix added to the INN. This approach to naming is under review, however, and the proposal being considered by WHO is that all biologics (not just biosimilars) should have a two-part name. The first part would be the INN, and the second part would be a unique four-letter identification code (“biologic qualifier” or BQ), distinct from the INN, to assist in identification of biologic substances for prescribing, dispensing, and pharmacovigilance [69].

Some countries have approved their own policies for biosimilar naming. According to the guidance issued by the Japanese regulatory Pharmaceuticals and Medical Devices Agency, the names of biosimilars should be easily distinguishable from those of the reference product and from those of other biosimilar products. Biosimilars are referred to by using the non-proprietary name of the reference product followed by the letters “BS,” in addition to the dosage form, dosage, and name of the manufacturer [73, 74]. Until January 2015, the naming convention for biosimilars followed by the Australian Department of Health Therapeutics Goods Administration for Australian Biological Names was similar to Japan’s in that it clearly distinguished biosimilars from reference products. Currently, however, the policy is under review in light of the proposed BQ system under consideration by WHO. In the interim, biosimilars will be identified by the Australian biologic name without a specific biosimilar identifier suffix [75]. The Mexican College of Rheumatology states that the label of each biologic product should clearly indicate whether it is an innovative biologic or biosimilar drug [55]. In Europe, approved biosimilars share the INN that corresponds with that of their reference product, and EMA advises that the commercial name, appearance, and packaging should differ. In the USA, the FDA recently issued draft guidance on the non-proprietary naming of biologics [76]. The proposed naming convention applies to all biologics, including originator products and biosimilars. Specifically, the FDA proposed that both reference products and biosimilars be given a four-letter suffix to the INN that is unique; the suffix selected will not have any special meaning. Although adoption of the WHO’s BQ system would be a voluntary decision by individual regulatory authorities, if widely accepted it would be a significant step toward global harmonization. Most authorities agree that a distinct brand name and/or a distinct non-proprietary name for a biosimilar are necessary to establish and maintain effective pharmacovigilance systems.

## Pharmacovigilance and risk management plans

Throughout the product life cycle, the ability to monitor and follow all biologics is critical to protecting patient safety. In order to do that, each biosimilar product should be readily identified and easily distinguished from the reference product and from other biosimilars. As mentioned above, pharmacovigilance describes the detection, assessment, and prevention of adverse effects after a product enters the market [26]. As with all biologics, appropriate post-marketing pharmacovigilance will be critical to the appropriate use of biosimilars. In general, regulatory agencies recommend that pharmacovigilance plans be developed and consider any known safety signals associated with the use of the reference product and its class [10–12, 45, 63].

Recent recommendations on how to ensure the safety and effectiveness of biosimilars in Latin America state that all Latin American countries should establish a certification program to train pharmacovigilance experts [77]. These experts would manage all aspects of data collection and analysis related to the use and safety of pharmaceuticals. Any additional specific safety monitoring or pharmacovigilance measures, including immunogenicity testing required for the reference biologic or its product class, should also apply to a biosimilar. In addition, any novel safety concerns that became apparent during evaluation of the biosimilar should also be evaluated. This underscores the importance of the well-defined pathways and regulations for the review, approval, and pharmacovigilance of biosimilars, factors that may be lacking for intended copy products.

Clinical safety of biologics, including biosimilars, must be monitored closely on an ongoing basis during the post-approval phase. There is a need to improve traceability, in particular with respect to individual batches or lots, to facilitate better identification and monitoring of post-approval safety issues [78]. Physicians and other health care providers need accurate data on adverse events associated with specific treatments to ensure the medicines they are prescribing are safe and effective. In addition, participation in registries with ongoing evaluation of safety in the clinical setting is often used to evaluate long-term safety. Registries are designed to understand the nature and frequency of adverse events and potentially identify risk factors in patient populations outside clinical trials. Naming of biosimilars may play a major role in registries, permitting identification of the exact product (originator or biosimilar) associated with a particular adverse event.

**Fig. 2:**
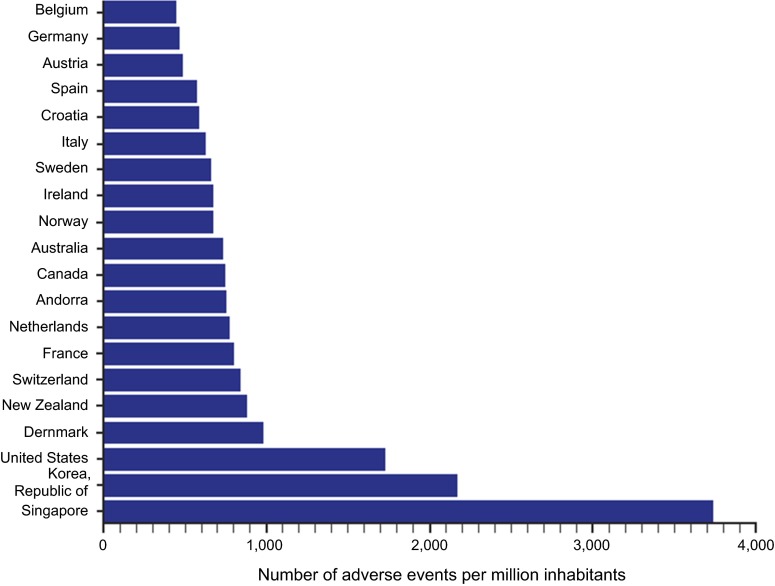
Annual number of adverse drug reactions reported to the World Health Organization from 2010 to 2015 (inclusive; per million inhabitants) [89]

Even with robust national reporting systems for pharmacovigilance, a serious limitation is the extent of underreporting of adverse events by health care providers. Wide variations in reporting rates of suspected adverse drug reactions (ADRs) have been found among countries. A survey of medical practitioners in nine European countries reported that the percentage of respondents in each country who stated they had reported an ADR at least once ranged from 19.4 % in Italy to 74.4 % in France [79]. National reporting rates obtained by direct inquiry of the national regulatory authorities in these countries showed a pattern similar to the individual practitioner findings (Table 4). An international system for monitoring ADRs was established in 1968 by WHO [80]. The Programme for International Drug Monitoring analyzes new adverse reaction signals from reports submitted by 120 countries, and today, the database contains more than 10 million reports of suspected ADRs [81]. Recent data collected worldwide indicate continuing disparity among countries in terms of reporting rates of ADRs. For example, among the 20 countries with the highest rates of reporting, the rates range from more than 3600 reports per million inhabitants per year in Singapore to approximately 400 reports per million per year in Finland (Fig. 2). Nonetheless, even in countries with high reporting rates, it has been estimated that fewer than 10 % of all adverse drug reactions are reported [82]. The success or failure of any pharmacovigilance activity depends on the timely and accurate reporting of suspected adverse reactions and proper traceability.

**Table 4:** Percentages of respondents who had reported a suspected adverse drug reaction (ADR) at least once to either a national agency or pharmaceutical manufacturer [79]

	Denmark	France	Ireland	Italy	The Netherlands	Portugal	Spain	Sweden	UK
Any ADR report	66.7	74.4	53.2	19.4	33.2	48.6	44.1	65.4	62.7
National reporting rate^a^	295.5	389.7	318.9	44.3	76.3	8.6	120.5	347.3	340.8

^a^Reports/10^6^ population/year

Risk management plans (RMPs) were introduced in 2005 in Europe to support a proactive approach in gaining knowledge on safety concerns through early planning of pharmacovigilance activities, and RMPs are indispensable in the pharmacovigilance of new drugs [83]. The objective of an RMP was to protect patients from adverse events by ensuring that the benefits of a medicine exceed its risks by the greatest margin possible [84]. RMPs use scientifically based methodologies to identify, assess, communicate, and minimize risk of a drug. RMPs include information on a drug’s safety profile, how its risks will be prevented or minimized, plans for studies to increase knowledge about safety and efficacy, and any known risk factors for development of adverse events. It has been recommended that the risk management plan for biosimilars focuses on heightened pharmacovigilance measures, identification of immunogenicity risk, and implementation and maintenance of post-marketing surveillance [85].

Evidence of safety and/or efficacy issues related to the use of intended copies in patients with RMDs and other conditions has emerged in Latin America and Asia. In a non-comparative observational study in 110 patients with rheumatoid arthritis, side effects were reported in 10 % of patients treated with Etanar^®^, an intended copy of etanercept marketed in Colombia [86]. Main causes of discontinuation of Etanar therapy were lack of efficacy, heart failure, nausea and dizziness, pneumonia, and asthma. In comparison, adverse events reported most frequently among patients treated with the originator etanercept include infections and injection site reactions [87]. In a second observational report of patients with rheumatic diseases from four hospitals in Mexico and Colombia, 10 of 14 patients treated with Etanar or Infinitam^®^ (another intended copy of etanercept) and 101 of 205 patients treated with Kikuzubam, an intended copy of rituximab, experienced at least one treatment-related adverse event [88]. Grade 3 or 4 adverse events were experienced by 14.3 % of patients receiving these intended copies. Other safety issues have been reported with recombinant insulin in Chile and epoetin alpha and beta in Thailand [19]. Because of the lack of rigorous pharmacovigilance for these drugs, including periodic safety update reports, it is difficult to know whether adverse events have gone undetected.

## Conclusions and recommendations

Biologics are vital to the management of patients with RMDs such as rheumatoid arthritis and other inflammatory and autoimmune conditions. By providing additional treatment choices, biosimilars may increase access to biologic therapies, which may improve patient care. Biosimilars have the potential to provide savings and efficiencies for health care systems, which can free up resources for other important aspects of health care. Increased access to biologics with the use of biosimilars must be accompanied by evidence of high quality and comparable safety and efficacy to the originator product. Biosimilars are developed using scientifically sound principles, and data requirements for the development and regulatory approval of biosimilars are much greater than those for small-molecule generic products. Intended copies of biologics are available in various countries, and the lack of information about efficacy and safety of these products compared with the originator biologic may put patients at risk. Products previously approved as intended copy biologic drugs should be evaluated according to the current regulations specific to those required for biosimilars and within a specified time period or be removed from market. High standards for global pharmacovigilance of non-originator biologics are essential and should include harmonization of biosimilar naming and superior systems for adverse event reporting and traceability of adverse events to specific products.
